# Systematic review of functional magnetic resonance imaging (fMRI) applications in the preoperative planning and treatment assessment of brain tumors

**DOI:** 10.1016/j.heliyon.2025.e42464

**Published:** 2025-02-06

**Authors:** Hamad Yahia Abu Mhanna, Ahmad Fairuz Omar, Yasmin Md Radzi, Ammar A. Oglat, Hanan Fawaz Akhdar, Haytham Al Ewaidat, Abdallah Almahmoud, Abdel-Baset Bani Yaseen, Laith Al Badarneh, Omar Alhamad, Laith Alhamad

**Affiliations:** a School of Physics, Universiti Sains Malaysia, USM, 11800, Penang, Malaysia; b Department of Medical Imaging, Faculty of Applied Medical Sciences, The Hashemite University, Zarqa, 13133, Jordan; c Physics Department, College of Science, Imam Mohammad Ibn Saud Islamic University (IMSIU), Riyadh, 13318, Saudi Arabia; d Department of Allied Medical Sciences-Radiologic Technology, Faculty of Applied Medical Sciences, Jordan University of Science and Technology, Irbid, 22110, Jordan; e Imagining Institute, Cleveland Clinic Abu Dhabi, Abu Dhabi, 112412, United Arab Emirates

**Keywords:** Brain tumor, Blood oxygenation level-dependent, Application, Apreoperative fMRI, Direct cortical simulation

## Abstract

The utilization of functional magnetic resonance imaging (fMRI) is critical in the preoperative planning phase of brain tumor surgery because it allows for a delicate balance between maximizing tumor resection and maintaining brain function. A decade of fMRI development was examined in this study, with a particular emphasis on its use in diagnosing and assessing the efficacy of brain cancer treatments. We examined the foundational principles, practical implementations, and verification of fMRI via direct brain stimulation, with particular emphasis on its capacity to detect cerebral regions affected by tumors that are eloquent in nature. Recently, fMRI has undergone significant progress, allowing for its integration into clinical workflows to facilitate precise mapping of brain functions. This extensive analysis encompasses the scrutiny of resting-state fMRI (Rs-fMRI) as a method of capturing functional connectivity, thereby providing significant insights into the management of patients with brain tumors. Methodological advancements, clinical applicability, and future orientations of fMRI are highlighted in this review, which emphasizes the substantial influence of the technique on neurosurgical planning and patient outcomes.

## 1 Introduction

Comprehending the role of the human brain as a central regulator of bodily functions is of utmost importance. This is due to the fact that the brain is considered to be one of the most crucial and intricate organs within the human body. Despite extensive scientific efforts spanning several years, brain tumors remain one of the most lethal forms of cancer. Patients with brain tumors exhibit a complex clinical situation due to localized structural changes in the brain caused by tumor growth as well as the broader consequences related to the mass of the tumor. However, the human brain has the propensity to adapt to pathological alterations in the neurological system. Malignancies in this particular case exhibit strong resistance to both traditional and innovative treatments because of the unique cell-intrinsic and micro-environmental features of brain tissues. Therefore, brain surgery is often regarded as a primary and conventional therapeutic approach for the management of brain malignancies [1,2].

Brain tumor surgery demonstrates complex issues because of the associated subtle tradeoff between the total removal of neoplastic tissue and the preservation of brain function [2,3]. Healthy or diseased human brain-defining activity can be studied using magnetic resonance imaging (MRI), fMRI or diffusion tensor imaging (DTI). MRI is a safe and non-invasive imaging technique that allows mapping of organs, tissues, and specific functions within the body. This method is applied for the diagnosis, detection of disease, and treatment monitoring of different ranges of disease conditions within the brain, abdomen, chest, and pelvis [4]. fMRI and DTI have emerged as promising tools for the non-invasive assessment of human brain function, as well as for determining the brain areas that must be spared to avoid functional impairment after surgery [3].

**Fig. 1:**
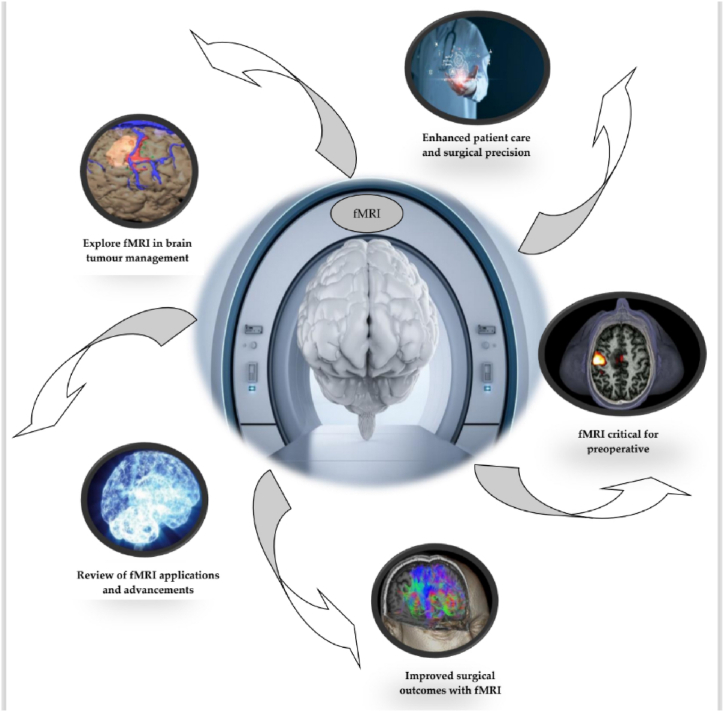
Graphical abstract. Integration of fMRI in Brain Tumor Management: A Visual Overview of Preoperative Planning and Treatment Efficacy. This dataset has been made available using appropriate standards and several visualizations highlighting different features in the dataset are presented in this work.

Over the past four decades, many techniques have been developed to map the functioning of the human brain. Two basic classes of mapping methods have evolved since then: (i) mapping, which localizes the underlying electrical activity of the brain, and (ii) mapping of metabolic consequences or the local physiology of altered brain electrical activity. Non-invasive neural electromagnetic methods, such as electroencephalography (EEG) and magnetoencephalography (MEG), are two techniques that form the former class of mapping. The two methods (EEG and MEG) allow exquisite time-based resolution of neural processes over a 10–100 ms time scale, and despite this, they appear with a poor spatial resolution of between one and many [4,5]. Among the latter classes of mapping methods, fMRI techniques have been used. This method has evolved over the years since the first successful experiment in 1990, a few years after MRI came into clinical application, and has continued to dominate the brain mapping field because of its low invasiveness, relatively wide availability, and absence of radiation exposure [6,7]. Fig. 1 shows the workflow in which the main procedures followed to generate the data described in this publication are highlighted.

This systematic review aims to investigate the applications of fMRI in the preoperative planning and treatment assessment of brain tumors, focusing on its contributions to enhancing surgical precision, preserving patient quality of life, and advancing our understanding of brain function. Additionally, the integration of fMRI with other imaging modalities and its limitations will be discussed to provide a holistic view of its clinical utility.

### 1.1 The functional magnetic resonance imaging (fMRI)

fMRI is employed as a non-invasive method to accurately identify and localize acute cognitive functions in the brain. This methodology has been widely employed to examine the human brain and its functional organization in both diseased and healthy individuals. The use of this instrument is highly regarded by clinical radiologists and investigators for examining task-related brain activity in patients with neuropsychiatric or neurological disorders. The utilization of this methodology has witnessed steady expansion as a clinical preoperative instrument for patients with brain cancer, providing valuable information that can significantly influence surgical decisions. Currently, fMRI plays a crucial role in the preoperative identification of eloquent brain regions affected by tumors. This information is invaluable to neurosurgeons as it aids in the development of an effective surgical approach [2,[8], [9], [10]].

fMRI is increasingly used for surgical planning and provides a non-invasive method for assessing the comparative eloquence of brain tumors. This technique is now actively used preoperatively in patients with brain tumors to evaluate functional relationships between the eloquent cortex and brain pathology. When used preoperatively, these methods can complement the surgical method to allow safe surgery and even determine the feasibility of the surgery. When a suitable task can be selected, the modality provides crucial supplemental information about the lesion-to-eloquence area proximity, which includes language and motor regions. In presumed tumor mapping in or near eloquent brain areas, such as language areas or the motor cortex, this technique can be advantageous in shortening the time of surgery, guiding the neurosurgical tactic, and even acquiring predictive information preceding surgery Smith [2].

For effective and safe neurosurgery, fMRI has been considered to provide a detailed preoperative assessment of the individual functional anatomy around a brain lesion. Hence, the importance of fMRI in managing brain tumor cases cannot be overemphasized. Recently, Vysotski et al. [11] conducted retrospective investigations to ascertain the differences in mortality and morbidity between patients with brain tumors who underwent fMRI preoperatively and those who did not. Compared to those without fMRI evaluation (no–fMRI group), enhanced mortality in the fMRI and fMRI plus Electrical Cortical Stimulation (ECS) groups was observed by both univariate and multivariate analyses, with tumor grade and age being the most significant influencers. Three years of survival benefits from the fMRI group were almost double those of the no-fMRI group. Atta Ul Aleem Bhatti et al. [12] utilized non-invasive fMRI in awake craniotomy in their attempt to excise low-grade gliomas (LGG) in young patients. The histopathology results revealed Grade II oligodendroglioma, while generic analysis confirmed no co-deletion at 1p/19q. The group concluded that mapping using non-invasive fMRI for intracranial space-occupying lesions is a crucial modality in a nation with limited resources, such as Pakistan. demonstrated the effectiveness of fMRI in the preoperative planning of brain tumor surgery when used to investigate high-grade glioma (HGG), LGG, and meningioma [2].

#### 1.1.1 Functional MRI methods

fMRI allows non-invasive imaging of regional brain functions, offering exceptional temporal and spatial resolutions. In addition, this technique allows the recording of signals from all regions of the brain, in contrast to EEG/MEG, which primarily captures signals from the cortical surface. In contrast to computed tomography (CT) or positron emission tomography (PET), fMRI can be used to investigate brain function non-invasive, safely, and effectively without the associated hazards of ionizing radiation. fMRI is typically performed with a spatial resolution of 3 mm^3^. This allows for precise measurement of blood flow alterations within the brain, even at a scale as small as 1 cubic millimeter. In contrast, CT scans typically assess changes in blood flow within a range of 5–10 cubic millimeters [13,14].

Furthermore, the system has recently been used to measure the hemodynamic response related to neural activity and has been similarly applied in studies of patients with neurodegenerative diseases, which is considered an additional technological application [5]. Several investigators have applied fMRI to identify abnormal functional brain activity during task performance in multiple patient populations, including those with demyelinating, neurodegenerative, cerebrovascular, and other neurological disorders [15,16]. This demonstrates the great potential of fMRI in basic and clinical studies [13].

The fMRI protocol can be sensitive to vicissitudes in regional blood perfusion and blood oxygenation, which results in neuronal activity or blood volume (for instance, by applying an injected magnetic resonance contrast agent). One fMRI method that is sensitive to blood oxygenation variables, known as blood oxygenation level dependent (BOLD, fMRI), allows an image spatial resolution of a few millimeters with a temporal resolution of a few seconds but is restricted by the hemodynamic response itself [[17], [18], [19]]. The potential of this method has been recognized since the early 1990s as MR is dissimilar to blood deoxyhemoglobin for functional brain imaging with MRI [5]. BOLD fMRI is a powerful method used to visualize the localization of cerebral activity in diseased and healthy brains. This method detects local increases in absolute blood oxygenation, which may be a direct consequence of neurotransmitter action, and therefore reflects local neuronal signaling.

### 1.2 BOLD functional MRI

It is widely recognized that the BOLD functional MRI system enables the localization of volumes on the scale of several cubic millimeters. BOLD-based fMRI has emerged as a prominent methodology for studying brain activity, encompassing both local and large-scale investigations [5,17,20]. This review aims to provide a comprehensive overview of the current state of research on the application of fMRI in the treatment of brain cancer. It discusses the general perspective, highlights the progress made in the past decade, and examines many applications of fMRI in this context. This study provides a comprehensive examination of the fMRI method, focusing particularly on its role in the preoperative planning for brain tumor surgery. It explores the historical development of fMRI and discusses recent advancements in its application in brain tumor therapies. In addition, a compilation of complete annual studies and discoveries on brain cancer mapping utilizing fMRI modalities for the period from 2011 to 2022 is required. The study additionally validated the instrument using direct cortical stimulation (DCS) and explored its prospective trajectory.

### 1.3 The application of fMRI tool: the journey so far

In clinical brain tumor management, fMRI has been widely used to plan surgical interventions and assess the risk of postsurgical functional deficits. This method is one of the most crucial tools for non-invasive special localization of brain function and is extensively applied to detect brain neural activity, including the visualization of eloquent cortical areas [1]. It was found that the fMRI localization of these eloquent brain cortical areas correlates firmly with invasive techniques, such as intraoperative ECS, which results in an increased extent of resection, surgical time reduction, and reduced craniotomy size [1,13].

#### 1.3.1 The application of fMRI tool in perioperative deficits

For preoperative planning, fMRI is extensively used in mapping eloquent brain areas to precisely predict perioperative deficits. Thus, the variable accounts of the precision of this method in language mapping were recorded. The technique has demonstrated high accuracy in evaluating hemispheric language dominance, helping brain surgeons assess the operative risk. In fact, for operative planning, this tool may be used as standard care in the diagnosis of patients with critical brain resection. Moreover, preoperative BOLD fMRI is performed in patients with intracranial lesions to enhance the surgical treatment [1,[21], [22], [23]].

#### 1.3.2 The application of fMRI tool in physiological effects of neuronal activity

Biomarkers are important in the diagnosis, treatment planning, and monitoring of disease progression in neurological diseases. Biomarkers can be genetic, proteomic, or imaging modalities. fMRI is one of the most important biomarkers, allowing for understanding brain activity and connectivity critical to preoperative planning and assessment of brain tumor treatment.

Moreover, in Alzheimer's disease (AD) [24], Parkinson's disease (PD) [25], multiple sclerosis (MS) [26], and other central nervous system diseases [27] that are under development as biomarkers, microRNAs have attracted interest for their potential to reflect disease status and therapeutic responses. By integrating these biomarkers (fMRI and microRNA) we can better understand neurological disorders and improve clinical outcomes.

#### 1.3.3 The application of fMRI tool in Alzheimer's disease

The fMRI approach has been utilized for the diagnosis, monitoring, prediction, and surgical management of various illnesses, including brain tumors, Alzheimer's disease, multiple sclerosis, epilepsy, stroke, Parkinson's disease, vascular malformations, trauma, and vegetative coma. The present methodology was employed to evaluate extensive evidence pertaining to neuroplasticity in multiple sclerosis. It reveals alterations in task conditions, resulting in modified patterns of deactivation or activation as well as the involvement of additional brain regions across all functional domains. The advent of RS-fMRI has enabled the examination of functional connectivity (FC), analysis of pertinent functional changes in individuals with multiple sclerosis across and within networks, and discovery of distinct resting state networks (RSNs) [28].

#### 1.3.4 The application of fMRI tool in eloquent mapping

Furthermore, fMRI has been used to assess brain function in diverse diseases such as multiple sclerosis. This method has shown that changed patterns of connectivity are used to recruit supplementary widespread eloquent brain networks better in tasks involving motor activity, memory, and sensory and cognitive functions than in normal controls [29,30]. fMRI tools are applied to localize eloquent neural responses evoked by motor, sensory, or cognitive tasks by measuring mapped changes in the oxygenation of blood hemoglobin that are activated by focal changes in neural activity. However, in this method, neuronal function is measured indirectly, non-invasively, well-tolerated by subjects, quickly acquired (even 20 min), and may provide wide eloquent brain tissue maps that could result in a post-treatment visual deficit. Active brain regions are indirectly detected depending on task-related changes in brain perfusion. BOLD signal changes may be detected by fMRI, which mostly results in an excellent spatial resolution of fMRI about ∼1 mm and involves deep locations [31].

#### 1.3.5 The application of fMRI tool in electrocorticography-BOLD fMRI

The present fMRI uses BOLD as the technique for evaluating active areas and, due to some acquired experience, the signals are not independently quantitative, though relative. Structural observation and understanding of which structures participate in certain functions have been granted by fMRI technology, which affords investigators the opportunity to advance their comprehension of brain structures [5,7,30,32]. A direct spatial correlation was recently observed in BOLD fMRI responses by using high-frequency band electrocorticography.

A combination of electrocorticography-BOLD fMRI revealed a spatial correlation between high-frequency band power and BOLD responses, providing evidence of supplementary agreement in temporal features. This combination has similarly confirmed the linear relationship between hemodynamics and neuronal responses in the primary motor and somatosensory cortices, indicating that high-frequency band power responses predict BOLD responses more accurately than stimulus timing alone [6,[33], [34], [35]]. In their studies, Gaglianese et al. [37] showed that BOLD fMRI spatial map activity in response to diverse types of sensory stimulation roughly corresponds to MEG and EEG responses in primary sensory cortices. Nonetheless, since MEG and EEG spatial resolution measurements are lower than those of fMRI, it is difficult to create a one-to-one neural and BOLD signal relationship.

#### 1.3.6 The application of fMRI tool in language function

Consistency in the preoperative fMRI mapping values for predicting postoperative decline in language function was objectively recorded. Specifically, Rosazza et al. [36] observed that preoperative language performance was responsible for 68 % of the alteration in the postoperative language outcomes, and fMRI language lateralization clarified 16 % more of it.

#### 1.3.7 The application of fMRI tool in neuroplasticity

It is noted that the early discovery of physiological location by fMRI, apart from conventional morphological assessment, permits an additional thorough study to monitor the tumor response. Furthermore, fMRI studies have identified processes related to neuroplasticity and compensatory hyperactivation. It is also being applied to investigate the modulatory effects of generic risk factors for neuronal disease on brain activation, including being used for diverse diagnoses as a prognostic biomarker of the disease course in addition to being used as a way to identify neural correlates of brain therapeutic interventions [29,37].

## 2 Methodology

### 2.1 Data collection

In a study we previously conducted titled Systematic Review Between Resting-State fMRI and Task fMRI in Planning for Brain Tumour Surgery [1], this review primarily addresses the use of Rs-fMRI and T-fMRI in the context of brain tumor surgery planning and their methodological differences. The clinical outcomes of Rs-fMRI compared with T-fMRI, with special emphasis on their individual contributions to the surgical planning process. While our current manuscript provides a comprehensive and expanded analysis of fMRI applications, it goes beyond simply comparing Rs-fMRI with T-fMRI but rather covers a range of functional MRI techniques used in the past 10 years for preoperative planning and evaluating the effectiveness of brain tumor treatments. Content includes basic ideas, practical applications, and validation techniques, with special emphasis on integrating fMRI into the clinical workflow. This review explores the historical development and notable advances in fMRI technology, examining its various uses in situations such as diagnosis, treatment monitoring, and functional connectivity analysis. In addition, it covers methodological advances and future directions for fMRI, and provides a comprehensive summary of its impact on neurosurgical planning and patient outcomes. It explores diverse clinical uses beyond surgical planning, such as studying neuroplasticity, assessing cognitive function, and evaluating treatment effectiveness. The research includes contemporary case studies and research findings demonstrating the practical utility of fMRI in various clinical situations. The submitted manuscript provides a comprehensive analysis of the importance of fMRI in brain tumor surgery, including a broader range of applications and technological advances compared to the previous paper [1]. The goal is to provide a comprehensive understanding of how fMRI can improve the comprehensive management of brain tumors, not only in surgical planning but also in treatment evaluation and beyond.

The PubMed/MEDLINE and Cochrane Library electronic databases were systematically queried by two diligent researchers to identify studies published between January 2012 and August 2022. The search terms employed included "brain," "brain cancer," "fMRI," "functional magnetic resonance imaging," "history," "advances," "BOLD," "blood oxygenation level-dependent," "application," "resting state," "preoperative fMRI," "task fMRI," "advanced fMRI," and "direct cortical stimulation." The preferred reporting items were mostly derived from guidelines for systematic reviews and meta-analyses as well as previous relevant research [38].

**Fig. 2:**
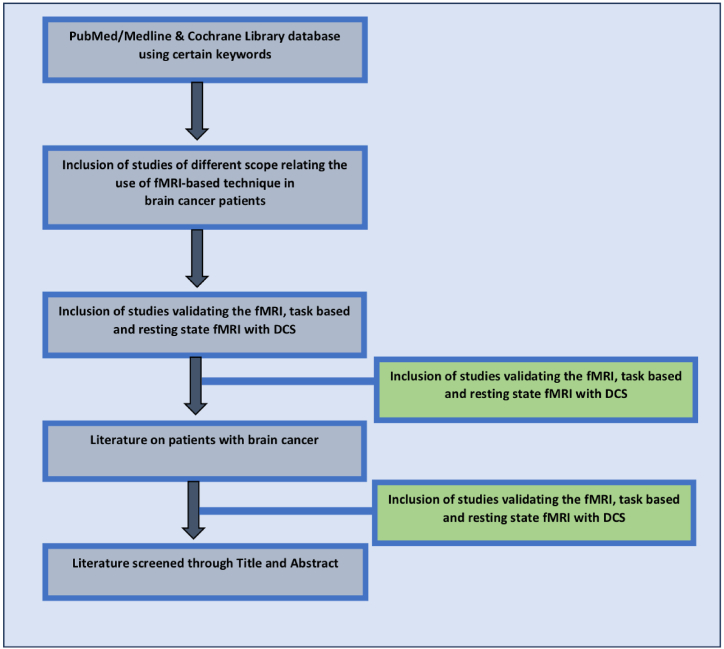
Flow chart displaying the selection process through which the studies were carried out.

**Fig. 3:**
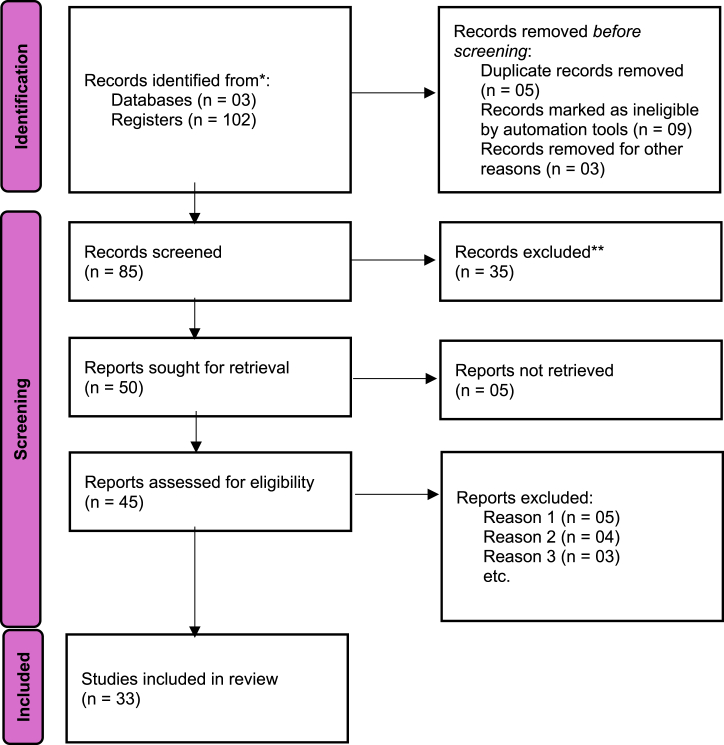
Identification of the studies through database and register.

The scope of this investigation was restricted to research on fMRI techniques, encompassing an examination of the overview, historical context, application, and advancements documented in the field of brain cancer imaging, primarily within the past decade. Furthermore, a thorough manual evaluation of the references cited in the studies obtained from the Scopus database was conducted. The research did not establish any limitations on language and publishing status; however, it primarily limited the publication date to the recent ten years, with a few exceptions. The researchers thoroughly screened all the entries in the final database, focusing on the titles and abstracts of each record. The eligibility of the final database was determined through a comprehensive process of consensus-based discussion. Fig. 2, Fig. 3 depict the flow charts illustrating the selection procedure employed for conducting the studies and the PRISMA flow diagram used for database and register searches, respectively. Table 1 provides a comprehensive overview of the study's outcomes.

**Table 1:** A collection of research analyzing brain function using fMRI.

NO	Study Location	Participants Number and Sex	Age	Handedness	Tumor Types	MRI Acquisition	Pre-Symptoms	fMRI Analysis	References
1	New York, USA	33 (19 male, 14 female)	48.9 ± 11 years	27 right-handed, 6 left-handed	Low-grade glioma (LGG), High-grade glioma (HGG)	3T scanner (Discovery 750W; GE Healthcare) with 24-channel head coils	Aphasia	Task-based language paradigms measuring language function before and after treatment, calculating the laterality index (LI)	[39]
2	Brigham and Women's Hospital, Harvard Medical School, Boston, USA	34 patients, sex not specified	Not specified	Not specified	Brain tumor	Movie-watching fMRI (mw-fMRI), task-based fMRI (tb-fMRI), resting-state fMRI (rs-fMRI)	Varying levels of language disruption	General linear model for tb-fMRI; Independent component analysis for mw-fMRI and rs-fMRI	[40]
3	Nanjing, China	176 (96 male, 80 female)	51.11 ± 13.74 years	Not specified	WHO II-IV gliomas	3.0-T MRI with 32-channel head coil, gradient-echo planar imaging sequence	Not specified in this summary	Regional Parameter of Resting-state fMRI-omics (RP-Rs-fMRI omics), with a comprehensive extraction of 420 features in 10 specific narrow frequency bins and three tumor parts	[41]
4	University Hospital Regensburg, Germany	19 patients (12 females, 7 males)	45.79 ± 19.63 years	Not specified	Glioblastoma multiforme, metastases, glioma, pilocytic astrocytoma, ependymoma, ganglioglioma, arteriovenous malformations, cavernomas, inflammatory tissue	Siemens Skyra 3-T with a 32-channel head coil	Visual field defects varied.	Eccentricity mapping for retinotopic analysis of the visual cortex	[22]
5	IRCCS Neuromed, Pozzilli, Italy	20 patients (M = 14, F = 6)	Mean: 44, Range: 23–77	Not specified	Glioma in peri-rolandic region	3T GE Signa HDxT scanner, T2∗-weighted EPI sequences	Motor functions	Comparing Finger Tapping Task (FTT) and Visual-Triggered Finger Movement Task (VFMT)	[42]
6	Shenzhen and Beijing, China	126 (72 male, 54 female)	42.21 ± 12.74 years	Right-handed	Low-grade gliomas (LGG), High-grade gliomas (HGG)	Siemens Verio 3.0T, GE-EPI sequence, 12-channel head coil	Not specifically mentioned.	Functional connectivity and topological features analysis using resting-state fMRI data, machine learning for grade prediction	[43]
7	Palermo, Italy	10 (7 male, 3 female)	25–67 years	9 right-handed, 1 left-handed	High-grade glioma (HGG)	3T MR scanner, T1-weighted 3D SPGR, resting-state fMRI acquired.	Language disturbance, seizure, headache, motor, and visual impairment	Seed-based functional connectivity analysis, identification of major functional connectivity networks before and after surgery	[44]
8	Houston, TX, USA	134 (85 successful tb-fMRI; 49 rs-fMRI processed; 28 males, 21 female)	47.5 years (17–78)	45 right, 2 mixed, 2 left	Various brain tumors, including glioblastoma and metastasis.	3 T GE MR750 scanner with an 8-channel head coil	Poor performance during tb-fMRI, too impaired to attempt tb-fMRI, weak/no BOLD activation near tumor, nonspecific BOLD activation, patient motion during tb-fMRI	Seed-based correlation (SBC) analysis when tb-fMRI was limited, successful in identifying functional language mapping in 84–88 % of cases evaluated by neuroradiologists.	[45]
9	The University of Texas MD Anderson Cancer Center, Houston, TX	20 patients (M = 14, F = 6)	Mean: 44, Range: 23–77	Not specified	Glioma in peri-rolandic region	Glioma in peri-rolandic region	Motor functions	Comparing Finger Tapping Task (FTT) and Visual-Triggered Finger Movement Task (VFMT)	[46]
10	Palermo, Italy	Not explicitly mentioned.	Not mentioned	Not mentioned	Not explicitly mentioned.	3T MRI, Discovery MR750w; General Electric Healthcare	Patients unable to cooperate with task-based fMRI.	Rest-fMRI to assess spontaneous BOLD signal fluctuation, identifying resting-state networks (RSNs) for surgical planning and postoperative evaluation	[20]
11	International Neuroscience Institute, Hannover, Germany	30 (16 male, 14 female)	18–77 years (mean 54 years)	Not mentioned	Supratentorial gliomas	3 T Scanner, T1 or T2 and rs-fMRI sequences	Neurologic deficits (e.g., impaired consciousness)	Seed-based connectivity analysis (SCA), identifying and analyzing resting-state networks (RSNs) in preoperative planning	[35]
12	Yuquan Hospital, Tsinghua University, Beijing, China	9 children (5 male, 4 female)	8–14 years (mean 11 ± 3)	Not specified	Tumors in the third ventricle	Philips 3.0 T Achieva TX MRI	Headache, nausea, vomiting	ReHo, ALFF, fALFF, DMN, and hippocampus connectivity changes post transcallosal interforniceal surgery	[47]
13	Karachi, Pakistan	1 male	43 years	Right-handed	Low-grade glioma (LGG)	3-T MRI, functional MRI (fMRI), BOLD imaging	Refractory seizures, walking difficulty	Noninvasive brain mapping, motor area mapping	[12]
14	Athens, Greece and TübingenGermany	69 (37 female, 32 male)	50 years (mean)	Right-handed	Glioblastomas, meningiomas, astrocytomas, oligodendrogliomas, hemangiomas, ependymoma, neurocytoma	1.5T Siemens Symphony-Vision scanner	Paresis, aphasia, finger tapping deficits.	Independent component analysis (ICA) of RS-fMRI to correlate motor and language functions with brain tumors	[48]
15	NIMHANS, Bengaluru, Karnataka, India	23 (16 male, 7 female)	38.9 ± 11.9 years	Right-handed	Tumors involving left IFG.	3.0-T Siemens Skyra MR scanner with 20-channel coil	Speech disturbances, headache, seizures, weakness	Language lateralization and speech centers analysis using picture naming task, BOLD imaging	[49]
16	University of Rochester, NY, USA	1 male (Patient AE)	26 years	Right-handed	Tumor in right temporal lobe	3T MRI, various fMRI tasks for mapping language and music processing	Deja vu, perceiving non-verbal sounds as voices.	Melody repetition task, behavioral and fMRI assessment of music processing, compared to language and other sound categories	[50]
17	Cologne, Germany	18 (10 male, 8 female)	50 ± 13 years	Not mentioned	Brain tumors adjacent to the corticospinal tract	3T MR scanner (MAGNETOM Trio, Siemens Healthcare)	Not specified, included motor deficits and epilepsy	Comparison of fMRI and nTMS for M1 seed volume delineation in DTI tractography	[51]
18	University Hospital Erlangen, Germany	60 (37 male, 23 female)	47.4 years mean.	Not mentioned	Various, including glioblastomas, anaplastic gliomas, low-grade gliomas, gangliogliomas.	1.5-T Siemens Sonata MRI with echo-planar imaging	Varied, including epilepsy.	Factual knowledge recall using fMRI, investigating retrieval deficits post-surgery in the parahippocampal gyrus	[52]
19	Montpellier, France	98 (55 male, 43 female)	40.5 ± 10.8 years	84 % right-handed	Diffuse Low-Grade Glioma	1.5T or 3T MR scanner with a 32-channel head coil	Not specified	Seed-based and independent component analysis (ICA) of resting-state connectivity, comparing with direct cortical stimulation	[53]
20	Toronto, Canada	18 (10 male, 8 female)	43.2 ± 13.7 years	Mostly right-handed	Low-grade glioma (LGG), High-grade glioma (HGG), and brain metastases	3T MRI, 8-channel head receiver coil	Speech and motor deficits in some patients	Test-retest reliability in motor and language tasks, spatial overlap, displacement of brain activity clusters, BOLD signal stability	[10]
21	Porto, Portugal and Leuven, Belgium	15 patients (12 men)	37.5 ± 12.4 years	7 left-handed; all left-lateralized for language	Brain tumors and epilepsy	Philips 3 T Achieva scanner with a 32-channel array head coil	Varied, including epilepsy and brain tumors.	Independent component analysis (ICA) for language network identification, verb-to-noun generation task for task-based mapping	[4]
22	Technische Universität München, Munich, Germany	35 (22 male, 13 female)	Not explicitly mentioned.	32 right-handed	Left-sided perisylvian brain lesions.	3-T MR scanner (Achieva, Philips Medical System)	Not specified, included aphasia in some patients	Combined noninvasive language mapping using rTMS and fMRI, compared with DCS during awake surgery	[54]
23	University of Wisconsin — Madison, USA	67 brain tumor patients (43 male, 24 female) and 25 vascular lesion patients (9 male, 16 female)	Brain tumor: average 48 years, Vascular lesion: average 43 years	Right-handed	Brain tumors and vascular lesions	1.5 or 3 T Sigma General Electric Healthcare MR imaging scanner	Not specified in detail	Task-dependent language lateralization, influenced by statistical threshold; expressive and receptive language tasks	[55]
24	Halifax, Nova Scotia, Canada	16 patients (9 female, 7 male)	39 ± 13 years	13 right-handed, 2 left-handed, 1 mixed handed.	Various, including anaplastic oligoastrocytoma, glioblastoma multiforme, oligodendroglioma, meningioma	4 T Varian INOVA scanner, MP-FLASH and two-shot spiral out sequences	Not specified in the abstract	ROC-reliability (ROC-r) analysis, optimization of preprocessing pipelines, spatial correspondence with cortical stimulation	[56]
25	Houston, Texas, USA	214 patients (110 male, 104 female)	18–74 years (median 44 years)	84 % right-handed	Intra-axial gliomas near eloquent cortex	1.5- or 3.0-T GE MRI scanners, EPI-based sequence	Motor or language deficits in 32 % of patients	Used in 40 % of cases for eloquent cortex localization; sensitivity and specificity in language and motor areas analyzed	[57]
26	Cologne and Duisburg, Germany	37 (19 male, 18 female)	16–78 years (mean 48)	Not mentioned	Glioblastoma, brain metastasis, astrocytoma, cavernomas, meningiomas, arteriovenous malformation, ependymoma	3T Philips Achieva MRI scanner	Hemiparesis, aphasia, seizures	Used for preoperative planning, identified motor and language areas, facilitated surgical strategy and decision-making	[58]
27	University Hospital Jena, Germany and Massachusetts General Hospital, USA	100 (52 male, 48 female)	25.2 ± 9.0 years	Not mentioned	Not applicable (study on healthy subjects)	3 T whole body MR scanner (MAGNETOM Trio Tim, Siemens)	Not applicable (healthy subjects)	mICA (masked independent component analysis) to identify intrinsic connectivity networks in the brainstem	[59]
28	University of Wisconsin, USA	49 (71 % male)	20–72 years (median 43 years)	Right-handed	Primary brain tumors in frontal, temporal, or parietal lobes	1.5 or 3 T GE Medical Systems MR scanner	Language deficits (various types of aphasia)	Task fMRI mapping of Broca and Wernicke areas, LI (lateralization index), and LAD (lesion-to-activation distance) analysis	[8]
29	Geneva, Switzerland	31 (20 male, 11 female)	10–58 years (mean 32 ± 14 years)	Right-handed (majority)	Medically intractable epilepsy, cerebral tumors	3T MRI, Magnetom Trio, Siemens, Erlangen, Germany	Epilepsy, brain tumors symptoms not specified.	8-min auditory semantic decision task, language area localization, functional connectivity analysis	[60]
30	Sosnowiec, Poland	58 patients (28 in fMRI group: 16 women and 12 men, 30 in control group)	Mean age 42.2 years in fMRI group, 47.8 years in control group	Not specified	Sensory-motor cortex tumors, including high-grade glioma, low-grade glioma, and metastatic tumor.	1.5 T S Avanto scanner with 8-channel matrix head coil	Not detailed in the abstract	Used BOLD (Blood Oxygenation Level Dependent) sequence for fMRI guided neuronavigation	[61]
31	Indianapolis, IN, USA	16 chemotherapy-treated (CTx+), 12 without chemotherapy (CTx-), 15 healthy controls	CTx+: 52.9 years, CTx-: 52.7 years, Controls: 50.5 years	Mostly right-handed	Breast cancer (stage 0, I, II, or IIIA)	1.5T GE Signa LX scanner, whole-brain coverage, gradient-echo echo-planar imaging	Not specified, post-surgery but before other treatments	n-back task to assess working memory during fMRI scans at baseline, 1-month post-chemotherapy, and 1 year later	[62]
32	Shanghai, China	23 (14 male, 9 female)	11–73 years (mean 46 years)	Not mentioned	Brain gliomas	Proton MRS, BOLD, and DTI scans before surgery	Headache, vomiting, seizure, limb numbness or weakness	Motor activation mapping, Cho/Cr and Cho/NAA ratio analysis, ADC and FA maps for tumor and peritumoral white matter tracts analysis	[63]
33	Larisa, Greece; Athens, Greece; Macon, GA, USA	87 (53 male, 34 female)	33–76 years (mean 62.8)	Not mentioned	Intracranial gliomas	1.5 T MRI scanner, fMRI with BRAVO pulse sequence, various motor, and language tasks	Not detailed	Motor and sensory mapping, language areas identification, comparison with intraoperative cortical stimulation (DCS)	[64]

## 3 Results

### 3.1 Applications of fMRI techniques in brain cancer studies

From a broad standpoint, fMRI provides novel insights into cognitive function in the human brain. The endogenous nature of the contrast agent renders it a less invasive neuroimaging technology than its predecessor positron emission tomography [7,17].

In the early stages of fMRI, the technique has been widely used as a guiding tool for brain tumor surgery, and is currently being used in a broad range of neurology-related studies from developmental to mental disorders, stroke, and dementia [1,7,13]. As a non-invasive method, fMRI has increasingly become a standard in the diagnosis of primary brain tumors, space-occupying brain lesions, and assessment of the extent to which cortical areas pertinent for particular functions, such as motor skills or language, can be considered in preoperative examinations. Eloquent cortical area visualization, surgical intervention planning, and assessment of the risk of postoperative functional deficits have been achieved using fMRI [1,22]. With these promising functions of fMRI in preoperative planning and clinical research, the method is not broadly used in day-to-day clinical practice because of factors such as limited accuracy, for example, delineating eloquent areas in single patients [13].

Recently, investigators have started to investigate spontaneous low-frequency (< 0.1 Hz) fluctuations in BOLD fMRI signals, referred to as RS-fMRI. This technique was introduced by Biswal et al., in 1995 [28]. Unlike TS-fMRI, low-frequency fluctuations in RS-fMRI signals were assessed while the patients were at rest, allowing the unearthing of functionally correlated cortical regions, even if they were removed anatomically. This leads to the detection of what is known as resting-state networks. Significant progress has been made in the use of this technique in clinical settings since the first functional mapping tests using BOLD fMRI were published in 1990 [28]. RS-fMRI has been recommended to reflect coherent networks in the visual, language, and somatosensory processing regions [19,48]. These inherent low-fluctuation networks persist, even though they are amended in states of reduced awareness, such as sleep or sedation. RS-fMRI represents a new prospect in advanced fMRI methods. It is a novel imaging method based on the quantification of hemodynamic changes after activation of brain areas [20,32].

This method has gained much interest as a potentially viable substitute for T-fMRI, particularly in neurologically or cognitively impaired subjects [65,66]. Appreciable information related to glioma-related functional brain detection can be obtained using resting-state fMRI. This technique may be helpful in uncooperative patients, such as young children, paralyzed patients, or patients with different mental statuses [38]. Compared with task-based fMRI, resting-state fMRI provides the added advantage of multiple network identification from the same scan [20].

**Fig. 4:**
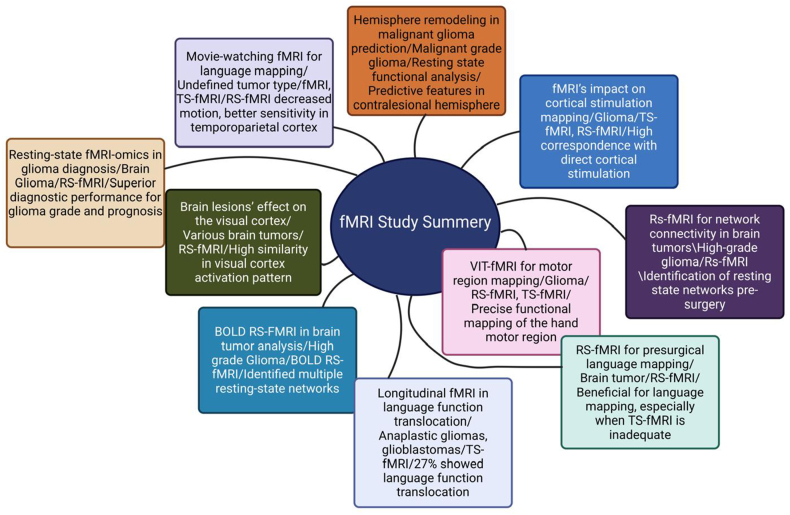
The findings of the content analysis of the studies included in this study. Created in BioRender. Abu Mhanna, H. (2025) https://BioRender.com/d98t083

Recently, some studies have reported the use of resting-state fMRI to effectively identify the functional networks underlying attention and speech/memory in patients with brain tumors, which may be helpful in planning surgery [22,38,67,68]. Moreover, RS-fMRI mapping of cognitive and emotional networks can be performed in individual patients. In some patients with brain and epilepsy, DCS has shown direct links between cognitive and emotional networks and their corresponding respective functions [69]. Liu et al. [41] reported a novel method called Regional Parameter of RS-fMRI-omics (RP-Rs-fMRIomics) by adopting an omics analysis strategy to RS-fMRI with exhaustible regional parameters, which demonstrated higher performance in predicting the prognosis of brain glioma, tumor grade, and IDH genotype compared to traditional single RS-fMRI. This novel approach represents an advancement in RS-fMRI clinical applications and contributes a new and novel imaging analysis for research in the brain tumor area. Table 2 and Fig. 4 compile recent investigations and the results (three studies per year) of brain function mapping using different fMRI techniques.

**Table 2:** Annual collection of recent investigations and findings of related brain cancer mappings using fMRI methods.

S/N	Scope of the investigations	Tumor and fMRI analysis types	Results/conclusion of the investigations	Reference
1	Longitudinal fMRI in language function translocation	Anaplastic gliomas, glioblastomas/TS-fMRI	27 % showed language translocation, related to Broca's area involvement.	[39]
2	Movie-watching fMRI for language mapping	Undefined tumor type/Mw- fMRI, TS- fMRI, RS-fMRI	Mw-fMRI shows decreased motion, sensitivity variation by cortex area.	[40]
3	Resting-state fMRI-omics in glioma diagnosis	Brain Glioma/RP-Rs- fMRI	Superior glioma grade, IDH genotype, and survival prediction with RP-Rs- fMRI.	[41]
4	Brain lesions' effect on visual cortex	Various brain tumors/TS-fMRI	High similarity in activation patterns, fMRI effective for retinotopic mapping.	[22]
5	ViTFMT for motor region mapping	Glioma in peri-rolandic regions/TS-fMRI	ViTFMT provides precise functional mapping, confirmed by electrical stimulation.	[42]
6	Hemisphere remodeling in malignant glioma prediction	Malignant grade glioma/Resting state functional analysis	Predictive functional features in contralesional hemisphere linked to glioma grade.	[43]
7	RS-fMRI for network connectivity in brain tumors	High-grade glioma/RS- fMRI	Identified resting state networks pre-surgery, potential for detecting post-surgery network changes.	[44]
8	nfMRI 's impact on cortical stimulation mapping	Glioma/TS- fMRI	High correspondence with direct cortical stimulation in identifying functional areas.	[45]
9	RS-fMRI for presurgical language mapping	Brain tumor/RS- fMRI	RS- fMRI useful in eloquent language region localization where TS- fMRI is inadequate.	[46]
10	BOLD RS- fMRI in brain tumor analysis	BOLD RS- fMRI in brain tumor analysis	Identified resting-state networks, useful for preoperative and postoperative analysis.	[20]
11	RS- fMRI with SCA in brain tumor effects on networks	Supratentorial gliomas/RS- fMRI SCA	Feasible in detecting high-level networks and their alterations in brain tumor patients.	[35]
12	Post-surgery brain function in young patients	Undefined tumor type/RS- fMRI	Recovery to pre-surgical state in brain function observed over time post-surgery.	[47]
13	fMRI in awake craniotomy for low grade gliomas	Low grade Glioma/TS- fMRI	Supports supratotal resection, showing less postsurgical morbidity and better outcomes.	[12]
14	RS- fMRI in pre-surgical function assessment	Various brain tumors/RS- fMRI ICA	RS- fMRI BOLD signal affected by tumors, correlates with language and motor function.	[48]
15	fMRI for speech centers in frontal gyrus tumors	Glioma/TS- fMRI	fMRI aids in identifying speech centers, differentiating based on speech capability.	[49]
16	fMRI in music processing of a right temporal lobe tumor patient	Low grade tumor/BOLD fMRI	Right superior temporal gyrus involved in melody processing, distinct from language processing.	[50]
17	Comparison of fMRI and nTMS in motor area mapping	Intracranial tumors/TS- fMRI	nTMS showed higher plausibility near tumor, indicating its effectiveness in motor mapping.	[51]
18	fMRI in memory function surgery	Gliomas/Gangliogliomas/TS- fMRI	Identified factual knowledge retrieval areas, assisting in avoiding memory deficits post-surgery.	[52]
19	RS- fMRI 's functional significance in language mapping	Low grade glioma/RS- fMRI (ICA).	Demonstrated higher resting state connectivity in language areas, aiding surgical planning.	[53]
20	TS- fMRI reliability in preoperative planning	Low and High Glioma/TS- fMRI BOLD	Found motor task activation more reliable, indicating choice of task affects fMRI reliability.	[10]
21	RS- fMRI vs TS- fMRI for language mapping	Glioma, Ganglioglioma, glioblastoma/TS-FMRI, RS- fMRI ICA	RS- fMRI showed good concordance with TS- fMRI in language mapping, with automated network identification.	[4]
22	RS- fMRI showed good concordance with TS- fMRI in language mapping, with automated network identification.	Left-sided perisylvian lesions/BOLD fMRI	Combined approach had high correlation to DCS, suggesting enhanced accuracy in language mapping.	[54]
23	fMRI in preoperative language and lesion impact	Various brain tumors/TS- fMRI	Found task type and threshold affect language lateralization, critical for preoperative planning.	[55]
24	fMRI reliability enhancement in preoperative mapping	Undefined tumor type/TS- fMRI	ROC-r analysis helped identify reliable datasets and optimize data processing for mapping.	[56]
25	fMRI's role in awake craniotomy	Intra-axial gliomas/task-based fMRI	Showed that fMRI was not significantly beneficial in identifying language areas compared to DCS.	[57]
26	fMRI for presurgical planning in brain lesion surgery	Brain lesions/Glioma/TS- fMRI	Consistent and rapid technique for localizing functional areas, aiding in surgical planning.	[58]
27	fMRI in brainstem activation and connectivity analysis	Undefined tumor type/TS- fMRI and RS- fMRI	Enabled identification of brainstem connectivity, showing high reproducibility in mapping. High reproducibility was exhibited by brainstem resting-state components across samples obtained at same MR scanner.	[59]
28	Language outcome and fMRI mapping in brain tumor surgery	High grade III and IV and low grade I and II tumors/TS- fMRI	Found correlation between tumor proximity to language areas and postoperative language deficits.	[8]
29	fMRI task development for language localization in surgery	Undefined tumor type/TS- fMRI	Fast fMRI language task localized crucial language areas, supporting surgery planning.	[60]
30	fMRI's role in reducing postoperative neurological deficits	Undefined tumor type/fMRI BOLD	Functional neuro-navigation reduced neurological deficits post-surgery, proving fMRI's utility in surgical planning.	[61]
31	fMRI in working memory changes related to breast cancer treatment	Breast cancer-related brain activation/TS- fMRI	Identified changes in brain activation associated with chemotherapy, providing insights into cognitive effects.	[62]
32	Multimodal fMRI in preoperative assessment of brain gliomas	Glioma/fMRI BOLD	Combining MRI BOLD and DTI provided comprehensive preoperative insights, aiding in glioma management.	[63]
33	fMRI and DCS mapping comparison in Intracranial Gliomas	Intracranial Glioma/TS- fMRI	High concordance between fMRI and DCS mapping, supporting fMRI's accuracy in preoperative sensory-motor mapping.	[64]

## 4 Discussion

### 4.1 Validation of fMRI with direct electrical cortical simulation

The goal of preoperative mapping is to localize functional brain regions proximate to the tumor, plan surgical methods, ascertain risks, and potentially render DCS needless [56]. Intraoperative DCS is generally considered the gold standard for functional cortical mapping. fMRI has traditionally been applied to localize functional brain areas by offering diverse tasks or stimuli that elicit neuronal responses, called task-based fMRI [[70], [71], [72]]. Task-based fMRI is widely used in mapping eloquent cortical sites before surgical procedures, but its accuracy in comparison with DCS has not been adequately reported, particularly for language-based functional regions [53,73]. In addition, fMRI BOLD contrast improvement as a non-invasive tool is continuously being applied to map the eloquent cortex in subjects with intracranial lesions, even though DCS is the gold standant [20].

Furthermore, many reported preoperative mapping findings using task-based fMRI of eloquent brain areas have been found to agree well with DCS (gold standard) with awake craniotomy [19,74]. Although DCS offers substantially high temporal and spatial resolutions, the method is time-consuming, invasive, and awake, depending on its validation [19].

Other studies have reported high specificity and sensitivity values of TS-fMRI compared to DCS and workout encouraging influence on preoperative decision making toward more belligerent approaches [53]. It is known that since RS-fMRI is largely centered on low-frequency detection of BOLD signals, the comparison between RS-fMRI and DCS could aid in clarifying the clinical significance of resting-state connectivity.

In addition, previous studies have highlighted the challenge of localizing language functions using resting-state fMRI. For instant, Joao Leote et [45] evaluated the impact of navigated task fMRI on the practical aspect of DCS and concluded that there is no influence of navigated task fMRI data on DCS in practice and that disturbance during the language presurgical procedure restricted the application of DCS mapping in awake surgery. Furthermore, 100/84 % correspondence between DCS and navigated task fMRI was observed in the identification of the precentral gyrus for motor function frontal inferior gyrus for language function. Genetti et al. [60] established a simple and short fMRI task that consistently localized crucial language areas in 35 patients with epilepsy foci/brain tumors who underwent resective surgery. They found that the proposed fast fMRI language procedure consistently localized the largely pertinent language areas in individual patients and that in five patients, the findings were concordant with electrocortical stimulation findings.

Stevens et al. [56] verified the utility of ROC-r analysis for reliable fMRI data set identification and found that the data sets with higher reliability indicated closer agreement with DCS, and poor fMRI data can similarly be identified by ROC-r at the time of scanning, giving room for repetition when needed. Kapsalakis et al. [64] evaluated the accuracy of preoperative fMRI in patients with intracranial glioma and compared it to DCS mapping. The results showed that, there was good concordance between fMRI and DCS pertaining sensorimotor cortex (91.9 %) and visual cortex (100 %). The Language mapping showed 85.4 % concordance, suggesting the indispensability of DCS mapping for language localization.

Ciavarro et al. [42] tested a new and creative cue-induced fMRI task called the visual-triggered finger movement task for accurate preoperative localization of the hand motor cortex compared to the traditional finger-tapping task. The new approach showed great potential in clinical FMRI and surgical management because of its precise identification of the hand-knob area, as well as good agreement with intraoperative direct electrical stimulation.

Trinh et al. [57] examined the role of fMRI as an intraoperative adjunct during awake craniotomy procedures and compared the results with those of DCS. Approximately 91 %, 93 %, and 100 % of sensitivity and 64 %, 18 %, and 100 % specificity in Broca's area, Wernicke's area, and motor areas, respectively. They concluded that the repetitive application of fMRI tools in language site identification is not valuable and more vitally follows awake craniotomy, practice tasks botched to avert neurological deficits [57].

### 4.2 Future trend of fMRI developments

The use of fMRI for preoperative planning and intraoperative management of brain surgery has been the subject of extensive discourse because of numerous studies. Numerous studies have demonstrated the reliability of fMRI in comparison to cortical stimulation for various purposes. However, several studies have highlighted certain limitations, particularly in the context of language localization [58,[75], [76], [77]]. Although there are currently no class I studies providing evidence for the efficacy of intraoperative or preoperative fMRI in neurosurgical procedures, it is worth noting that numerous neurosurgical institutions have performed fMRI scans prior to brain tumor surgeries in close proximity to functional areas. Therefore, it is imperative to conduct a thorough examination and assessment in order to determine whether fMRI truly provides additional preoperative insights that can impact the surgical approach and assist in intraoperative decision-making for brain tumor surgery [58].

Even though sufficient information is being obtained regarding the distribution of human language functions and related networks, localization/mapping of this function using fMRI tools remains a challenge and needs to be further investigated for enhancement. Assessing functional cortical organization using non-invasive techniques is crucial, and fMRI is the standard method that has been applied, especially in preoperative planning, for non-invasive mapping of motor and language functions. However, this method has yet to provide reliable language mapping results that exhibit only a slight correlation with intraoperative DCS. Recently, a group of researchers suggested that a combination of fMRI and other non-invasive methods can lead to a higher correlation with DCS. Ille et al. [54] combined navigated transcranial magnetic stimulation with fMRI in their work, and the findings presented a higher correlation to DCS compared to when they were used alone. For neurophysiological evaluations, a more frequent combination of fMRI with other related methods is noted [78]. In fact, the latest breakthrough in fMRI assessment has enabled close monitoring of changes in tumor physiology. In addition to morphological changes, fMRI monitors therapeutic reactions and detects persistent tumors by measuring changes in the physiology of the tumor microenvironment [78].

Furthermore, personalized fMRI can effectively capture functional regions near and within tumors. Cui et al. [79] developed an appealing approach called the individual functional network parcellation method using RS-fMRI that magnificently delineated functional regions in nearby and within brain tumors. It was found The tumoral mass (33.2 % of it) was observed to be functionally active and also showed robust functional connectivity with nontumoral brain networks. In general, the findings show that the method can successfully capture and delineate functional networks in nearby and within brain tumors. This approach was noted to have huge clinical application potential in patients undergoing resection. More recently, Posse et al. developed advanced imaging technologies, such as real-time fMRI, that allow neurosurgeons to make vital decisions in the surgery room. The group is developing turbo functional imaging in real-time tagged TurboFIRE to localize brain activity during an ongoing scan (fMRI) to enable physicians to map the exact area of the eloquent cortex-like brain regions with specific functions to support brain cancer operations [80].

## 5 Conclusion

Managing patients with brain tumors is difficult because tumor growth causes local anatomical changes in the brain, as well as mass effects. Adaptation of the human brain function to pathogenic nervous system alterations is common. Preoperative neurophysiological investigations for the surgical resection of functional areas/networks are crucial. Many independent studies have examined fMRI during preoperative planning of tumor surgery. fMRI, a non-invasive method for measuring brain tumor eloquence, has also garnered attention in presurgical planning. Review of fMRI use in brain tumor treatment and new advances over the last decade. fMRI has extensively studied brain neural activity detection. Clinical radiologists and investigators use fMRI to evaluate task-related brain activity in patients with neurological or neuropsychiatric disorders, without invasiveness. Since its introduction in the early 1990s, this approach has garnered clinical attention owing to its ability to study the human brain and its functional organization in diseased and healthy people. Over the last decade, fMRI, DCS, and future brain-cancer mapping studies have been reviewed annually. Comparison and subjective discussion of fMRI tool accuracy with DCS in eloquent cortical regions. Over the past decade, fMRI and DCS have shown significant concordance in mapping motor functional areas; however, language-based functional regions are difficult to localize. The technique may be developed to combine fMRI with other non-invasive tools to improve DCS correlation. Navigated transcranial magnetic stimulation and diffusion tensor imaging tractography have been used with fMRI to improve the DCS correlation.

## CRediT authorship contribution statement

**Hamad Yahia Abu Mhanna:** Writing – review & editing, Writing – original draft, Visualization, Validation, Supervision, Software, Resources, Project administration, Methodology, Investigation, Funding acquisition, Formal analysis, Data curation, Conceptualization. **Ahmad Fairuz Omar:** Writing – review & editing, Writing – original draft, Supervision. **Yasmin Md Radzi:** Writing – review & editing, Supervision, Methodology. **Ammar A. Oglat:** Writing – original draft, Validation, Formal analysis. **Hanan Fawaz Akhdar:** Writing – review & editing, Writing – original draft, Supervision, Funding acquisition. **Haytham Al Ewaidat:** Writing – review & editing. **Abdallah Almahmoud:** Visualization, Methodology, Data curation. **Abdel-Baset Bani Yaseen:** Writing – review & editing, Visualization. **Laith Al Badarneh:** Writing – original draft, Methodology. **Omar Alhamad:** Visualization. **Laith Alhamad:** Writing – review & editing, Methodology.

## Data availability statement

No additional data was used for the research described in the article.

## Funding statement

This research did not receive any specific grant from funding agencies in the public, commercial, or not-for-profit sectors.

## Declaration of competing interest

The authors declare that they have no known competing financial interests or personal relationships that could have appeared to influence the work reported in this paper.
